# Unveiling the Uncommon: Pyoderma Gangrenosum as an Extraintestinal Complication of Ulcerative Colitis

**DOI:** 10.14309/crj.0000000000001675

**Published:** 2025-04-16

**Authors:** Caleb Glover, Ali Rida, Eric Nguyen, Edward Cay, Gisela Ocasio-Cortes, Summer Stefanko, Arman Fotouhi, Albert Ross

**Affiliations:** 1 McLaren-Greater Lansing Hospital, Lansing, MN; 2 Michigan State University, East Lansing, MN

**Keywords:** pyoderma gangrenosum, ulcerative colitis, inflammatory bowel disease

## Abstract

Pyoderma gangrenosum (PG) is a rare inflammatory skin disorder characterized by neutrophil accumulation, commonly appearing as erythematous papules or pustules that can coalesce into extensive ulcers. It is the second most common dermatologic manifestation of inflammatory bowel disease, though it affects <1% of patients with inflammatory bowel disease. We present a case of a 66-year-old woman with a history of hypothyroidism and hypertension who developed nonhealing abdominal wounds following a small pustule. Despite multiple debridements and antibiotics, her condition worsened, alongside a history of bloody diarrhea for 2.5 years. Investigations led to a diagnosis of ulcerative colitis and PG. The patient was treated with high-dose steroids and infliximab. This case highlights the rarity of abdominal PG as an extraintestinal manifestation of ulcerative colitis and emphasizes the importance of early diagnosis and treatment in improving outcomes for affected patients.

## INTRODUCTION

Pyoderma gangrenosum (PG) is a rare neutrophilic dermatosis that often starts as a pustule and can turn into a deep ulcer and eventual necrosis.^1^ It is a common cutaneous manifestation of inflammatory bowel disease (IBD), affecting 5%–20% of patients with ulcerative colitis and often presents during an exacerbation of ulcerative colitis.^2^ Although PG itself is not uncommon in ulcerative colitis, PG presenting on the abdomen without prior trauma or a stoma is unusual. The condition is challenging to diagnose due to its varied presentations but also poses difficulties in management, especially when it manifests in atypical locations such as the abdomen. The clinical features of PG can vary widely, and its diagnosis often requires a thorough evaluation to exclude other potential causes of ulceration, including infections, malignancies, and other inflammatory conditions.^3^ In this study, we present a rare case of a 66-year-old woman with PG on the abdomen in the absence of trauma or stoma.

## CASE REPORT

A 66-year-old woman with a history of hypothyroidism and hypertension presented with longstanding gastrointestinal symptoms, including progressively worsening bloody diarrhea over 2.5 years, which led her to seek care for nonhealing abdominal wounds. The wounds began 7 weeks before presentation with a small pustule, which she attempted to pop, progressing into a large wound despite multiple debridements and antibiotics. Her symptoms included increased pain, erythema, and worsening ulceration.

Despite receiving surgical debridement from the general surgery team, her wound showed minimal improvement and she was placed on a 7-day course of oral antibiotics, including trimethoprim/sulfamethoxazole and cephalexin. After these procedures, her symptoms persisted, leading to increased pain and erythema surrounding the wound.

On her presentation to the emergency department, her physical examination showed notable tenderness around her surgical site, where the left abdominal wound appeared necrotic and exposed underlying adipose tissue. A smaller wound was noted in the right lower quadrant, with the left abdominal wound measuring approximately 8 cm in diameter. Laboratory tests indicated an elevated white blood cell count of 12.3 × 10^3^/µL and a C-reactive protein level of 4.5 mg/dL (normal C-reactive protein < 1 mg/dL), suggestive of an inflammatory process.

A computed tomography scan of the abdomen and pelvis, performed shortly before her admission, revealed no acute intra-abdominal or pelvic abnormalities, ruling out the presence of an abscess and identifying the issue as an abdominal wall infection. She underwent a colonoscopy, which detected circumferential and continuous inflammation, extending from the anal canal to the cecum. The terminal ileum was not intubated. Pathology reported ulcerative colitis, with biopsies revealing both chronic active colitis characterized by ulceration with crypt abscesses in the rectosigmoid region, and a cecal polyp diagnosed as a tubular adenoma.

**Figure 1.**
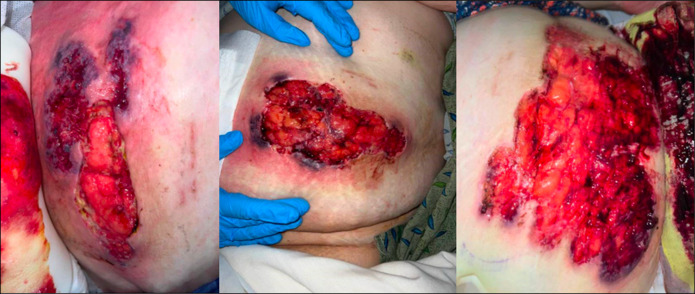
Images above depict the wounds the patient initially presented to the emergency department with. The wounds were large, deep ulcerations with distinctive violaceous (purple-red) borders with undermined edges classically seen in pyoderma gangrenosum.

Given the diagnosis of PG, confirmed by the skin biopsy which showed dense neutrophilic infiltration without vasculitis or immune deposits, the treatment plan during her hospital stay involved continuance of broad-spectrum intravenous antibiotics (cefepime and metronidazole) to manage her abdominal wall infection. Oral mesalamine was initiated, improving IBD symptoms. Given her PG diagnosis, she was started on a high-dose corticosteroid regimen, beginning with prednisone at 60 mg daily. She later received outpatient infliximab therapy (Figure 1).

## DISCUSSION

### Pathophysiology

The exact etiology of PG remains unclear, but it is believed to involve an aberrant immune response characterized by neutrophil accumulation and dysregulation of cytokine production.^4^ High levels of interleukin-8 have been detected in the skin lesions of patients with PG, suggesting a role for this chemokine in the pathogenesis of the disease.^4^ In addition, the involvement of T cells and macrophages has been implicated in the inflammatory process, indicating that PG may represent a complex interplay between innate and adaptive immune responses.^5^ The relationship between PG and ulcerative colitis is particularly noteworthy, as PG can develop independently of bowel disease activity, complicating the clinical picture since patients may experience skin manifestations without concurrent gastrointestinal symptoms.^6^ Understanding the pathophysiological mechanisms underlying PG in the context of IBD is essential for developing targeted therapeutic strategies.

### Diagnostic challenges

Diagnosing PG can be particularly challenging due to its clinical similarity to other ulcerative conditions. Histopathological examination is often necessary to differentiate PG from other causes of ulceration, such as infections or vasculitis.^3^ The absence of vasculitis and immune deposits in biopsy specimens supports a diagnosis of PG, while the presence of dense neutrophilic infiltration is a hallmark of the condition.^7^ However, the diagnosis is often made clinically based on the characteristic appearance of the lesions and the patient's history of underlying systemic disease.^3^ In the case of the aforementioned patient, a thorough investigation, including a wound biopsy and imaging studies, was crucial in establishing the diagnosis of PG as an extraintestinal manifestation of ulcerative colitis.

### Treatment strategies

The management of PG typically involves systemic corticosteroids, which are considered the first-line therapy due to their anti-inflammatory properties.^5^ In cases where corticosteroids are insufficient or contraindicated, biologic agents such as infliximab and adalimumab have shown efficacy in managing PG associated with IBD.^5,8^ The patient's treatment regimen included high-dose steroids and mesalamine for her ulcerative colitis, followed by outpatient infliximab therapy, which is consistent with current management guidelines.^5,7^ Recent studies have also explored the use of other immunosuppressive agents, such as cyclosporine and mycophenolate mofetil, in treating refractory cases of PG.^9^ Surgical debridement of ulcers is generally contraindicated, as it can exacerbate the condition and lead to further necrosis.^10^ Therefore, a careful and individualized approach to treatment is essential for optimizing patient outcomes.

PG represents an uncommon but significant extraintestinal complication of ulcerative colitis, particularly when it presents in atypical locations such as the abdomen. Clinicians must be vigilant in recognizing this condition, as early diagnosis and appropriate treatment can markedly improve patient outcomes. The case presented in this study illustrates the importance of maintaining a high index of suspicion for PG in patients with IBD, particularly those exhibiting nonhealing wounds or unusual dermatological manifestations. Further research is needed to elucidate the immunological mechanisms and optimize treatment strategies for atypical PG, ensuring improved patient care.

## DISCLOSURES

Author contributions: A. Rida contributed case summary section; S. Stefanko and A. Fotouhi contributed to the introduction; E. Nguyen contributed to writing case and editing; E. Cay provided editorial assistance; G. Ocasio-Cortes helped with editing and writing; A. Ross was the attending and contributed both writing and editing the paper. C. Glover is the article guarantor.

Financial disclosure: None to report.

Previous presentation: ACG Annual Scientific Meeting, Philadelphia, PA, October 29, 2024.

Informed consent was obtained for this case report.
